# Artifact removal by template subtraction enables recordings of the frequency following response in cochlear-implant users

**DOI:** 10.1038/s41598-024-56047-9

**Published:** 2024-03-14

**Authors:** Robin Gransier, Robert P. Carlyon, Matthew L. Richardson, John C. Middlebrooks, Jan Wouters

**Affiliations:** 1 ExpORL, Department of Neurosciences, Leuven Brain Institute, KU Leuven, Leuven, Belgium; 2 Cambridge Hearing Group, MRC Cognition and Brain Sciences Unit, University of Cambridge, Cambridge, UK; 3 Department of Otolaryngology, University of California at Irvine, Irvine, CA USA; 4 Center for Hearing Research, University of California at Irvine, Irvine, CA USA; 5 Departments of Neurobiology and Behavior, Biomedical Engineering, Cognitive Sciences, University of California at Irvine, Irvine, CA USA

**Keywords:** Auditory system, Midbrain

## Abstract

Electrically evoked frequency-following responses (eFFRs) provide insight in the phase-locking ability of brainstem of cochlear-implant (CI) users. eFFRs can potentially be used to gain insight in the individual differences in the biological limitation on temporal encoding of the electrically stimulated auditory pathway, which can be inherent to the electrical stimulation itself and/or the degenerative processes associated with hearing loss. One of the major challenge of measuring eFFRs in CI users is the process of isolating the stimulation artifact from the neural response, as both the response and the artifact overlap in time and have similar frequency characteristics. Here we introduce a new artifact removal method based on template subtraction that successfully removes the stimulation artifacts from the recordings when CI users are stimulated with pulse trains from 128 to 300 pulses per second in a monopolar configuration. Our results show that, although artifact removal was successful in all CI users, the phase-locking ability of the brainstem to the different pulse rates, as assessed with the eFFR differed substantially across participants. These results show that the eFFR can be measured, free from artifacts, in CI users and that they can be used to gain insight in individual differences in temporal processing of the electrically stimulated auditory pathway.

## Introduction

The cochlear implant (CI) is considered to be the most successful sensory neuroprosthesis. It can restore speech reception by stimulating the auditory nerve with electrical pulse trains that convey the temporal envelope of a limited number of spectral channels^1^. The CI, however, performs poorly in conveying the fundamental frequency (F0) of the human voice, thereby depriving CI users of pitch perception^2^, gender identification^3^, prosody (how we say something)^4^, and understanding of tonal languages^5^. Furthermore, being able to perceive F0 and F0 differences is beneficial when listening in challenging listening conditions^6^.

A number of CI stimulation strategies have been developed to improve F0 encoding, either by explicitly enhancing or aligning the F0 modulations across stimulation channels^7–13^, or by encoding F0 in the stimulation channels that stimulate the apical regions of the cochlea^14,15^. Nevertheless, the overall success of these stimulation strategies is limited. It is hypothesized that one of the attributing factors to this limited success is the inability of the stimulated neurons to encode F0, which is associated with neurodegeneration due to etiology and long-term hearing loss^16^. The degree of neurodegeneration and its functional impact can vary substantially across CI users (see Schvartz-Leyzac et al.^17^ and Pfingst et al.^18^ for an overview). Another possibility is that conventional CIs are unable to optimally stimulate the neural ensembles in the cochlea that are specialized in the encoding of F0-relevant rates^19^. That is, CI users are able to perceive pitch based on rate cues^20–24^ only up to around 300 pulses per second (pps)^24^, compared to rate-pitch sensitivity up to 700–800 pps in normal-hearing listeners. The upper limit of rate-pitch sensitivity can vary widely across CI users and across the CI array within individuals^20–23^. Insight in the neural basis of this variability could pave the way for the development of future CI electrode designs and neural-inspired stimulation strategies that give CI users better access to F0 cues.

The frequency following response (FFR), which is a phase-locked electrophysiological response to the F0 of a sound (e.g., such as a tone, a vowel, or a pulse train^25^), has the potential to characterize the biological limitations of rate encoding, noninvasively, at the level of the brainstem. This response synchronized to stimulus pulses, as measured with EEG, originates predominantly from the brainstem when evoked with rates ≥ 100 Hz, and its strength has been associated—in the acoustically-stimulated auditory pathway—with pitch perception^26–28^ and speech perception in noise^29^.

Measuring the electrically-evoked FFR (eFFR) in CI users is, however, challenging as the electrical-stimulation artifacts inherent to CI stimulation contaminate the EEG recording^30^. The process of isolating the stimulation artifact from the neural response is difficult when measuring phase-locked activity to a pulse sequence, as both the response and the artifact overlap in time and have similar frequency characteristics^31^. Although various artifact-removal methods have been applied successfully to measure phase-locked responses in CI users^32–37^—e.g., the envelope following responses evoked with amplitude modulated pulse trains–, applying these methods to the eFFR is not straightforward. Most artifact-removal methods are based on a priori assumptions about both the artifact and response waveform^35,36^. Currently there is, however, only limited knowledge about details of the eFFR waveform evoked in CI users and, so far, only one study has been reported in the literature that attempted to measure eFFRs to pulse rates > 100 pps in CI users. In that study, Gransier et al.^30^ removed the stimulation artefacts by blanking the measured waveform across the time of the artifact and then making a linear interpolation across the blanked samples. Although this method is highly effective for obtaining artifact-free envelope following responses evoked with amplitude-modulated pulse trains^32,37–40^, it can heavily distort the eFFR waveform when evoked with an un-modulated pulse train^30^ and is therefore of limited use for measuring artifact-free eFFRs in CI users.

In contrast to artifact removal methods based on blanking and linear interpolation, template subtraction has the potential to remove the stimulation artifact while leaving the neural response intact^33,41^. In template subtraction, an artifact template is constructed and then subtracted from the EEG signal that contains both the artifact and the neural response. State-of-the-art template subtraction methods are, however, often based on: (i) additional measures that either contain no neural response; or on (ii) alternating-polarity stimulation^33,42–45^ (i.e., the recorded artifacts have an in-opposite phase when stimulated with the different polarity stimulation). Whereas the former is difficult to obtain when the artifact model is based on the same stimulation characteristics as used to evoke the eFFR, in the latter, the neural response to the different polarities can differ^41,46–48^. An alternative approach is to base the artifact template on an a priori defined artifact model. Although this approach works well when applied to simple neural responses^44,45,49^, it becomes more challenging to successfully apply this method when the template is fitted to a recording that also contains a complex overlapping neural response, as is the case with the eFFR.

Here we introduce a new artifact removal method that constructs an artifact template of the artifact tail directly from the EEG recording (i.e., containing both the stimulation artifact and the neural response) by implementing a neural-response neutralization step prior to the construction of the artifact template. We used this new artifact template subtraction with recordings of eFFR to evaluate non-invasively the phase-locking ability of the brainstem of CI users to pulse rates ranging from 128 to 303 pps. The measurements yielded detailed information about the rate-dependent phase-locking ability at the level of the brainstem of the electrically-stimulated human auditory pathway of CI users.

## Methods

### Participants

The development and testing of the artifact subtraction method was based on two datasets, both obtained with patients implanted with CIs made by the Cochlear company. Participants had either a CIC3 or CIC4 pulse generator in their implant. The first dataset (DS1), contained newly gathered data from four subjects, whereas the second dataset (DS2) contained the data of three CI users reused from Gransier et al.^30^. The participating CI users (see Table 1) were young adults, selected because they were most likely to have robust eFFRs^50^. An exception to this was participant S7 who was 69 years of age and who was not expected to have a measurable eFFR to any of the rates used here. Her data were included to assess the effectiveness of the artifact removal method when no neural response is present.

**Table 1:** Participant details.

Subject	Implant	Age (years)	Implant use (years)	Tested ear	Etiology
S1	CI522	27	4.5	Right	Progressive
S2	CI622	26	0.5	Left	Progressive
S3	CI422	26	8	Right	Progressive
S4	CI622	24.5	22.5	Right	Congenital
S5	CI522	43	2.5	Left	Hereditary
S6	CI24M	24	22.5	Left	Meningitis
S7	CI24RE	69	7.5	Right	Unknown

The studies in which the data was collected were conducted at ExpORL, KU Leuven (participant S1-S4, S6, and S7) and Cambridge, U.K., (participant S5). The studies were approved by the Medical Ethics committee of the University Hospital in Leuven (UZ Leuven) (approval number: B32201941114) and the National Research Ethics Committee of the East of England (ref. number 00/327). All methods were carried out in accordance with the relevant guidelines and regulations—declaration of Helsinki. Written informed consent was obtained from all participants before testing.

### Stimulation

The stimulation sequence used to evoke the eFFRs was a 2.024 s epoch of pulse rate **A** followed by a 2.024 s epoch of pulse rate **B**. This pattern was then continuously repeated, and the number of stimulated epochs depended on the protocol and the subject, but a minimum of 384 epochs per pulse rate was obtained from each subject (Supplementary Table I). In DS1, three A-B combinations were stimulated: 128 and 233 pps, 163 and 268 pps, and 198 and 303 pps (yielding data at 128, 163, 198, 233, 268, and 303 pps). In DS2, pulse rate **A** was fixed at 94 pps and pulse rate **B** was either 128, 162, or 196 pps. Note that the pulse rate of 94 pps is not included in the present analysis as this neural response is expected to be evoked by neural generators, including at the thalamocortical level, and therefore does not isolate the responsiveness of the brainstem generators^30^. This approach of alternatively presenting the pulse rates was used to minimize the effect of loudness adaptation, which—as we noticed during pilot experiments—can occur when the same pulse rate is presented continuously. A trigger was sent to the EEG system at the start of each combination of **A** and **B** epochs to sync the electrical stimulation with the EEG recording.

The neural ensembles of the auditory nerve were stimulated with the most apical electrode (electrode 22) in monopolar mode, with combined casing and extra ball electrode serving as the return electrodes. The pulse trains consisted of cathodic-first biphasic pulse trains, with a phase width of 36 and 25 µs for respectively DS1 and DS2, and an interphase gap of 8 µs. The stimulation levels were set at the maximum comfortable loudness level (MCL), which was determined at the start of each experiment by using a seven-point categorical loudness scale (i.e., “inaudible”, “very soft”, “comfortable loud”, “loud”, “very loud”, and “unbearable”); the MCL was set at the last level before it was perceived as “very loud”. For DS1, only rates of 128, 198, and 303, were assessed and the stimulation levels of the other pulse rates were interpolated based on the obtained MCL levels. For DS2, the MCL level was determined for the 94-pps pulse train and the other pulse rates were loudness balanced to this pulse rate by using a two-down one-up loudness balancing procedure (for more details about DS2 see Gransier et al.^30^).

Stimuli were generated in Matlab^51^ and were sent to the CI via a research interface. The research interface consisted of a computer with custom-written software that interfaced with the Nucleus Implant Communicator (i.e., either NIC3 of NIC4) and was connected to the CI by means of a programming device and a research processor (i.e., either the L34 or the CP900). The Nucleus Implant Communicator, programming device, and research processors were provided by Cochlear Ltd.

For the CIC4 implant stimulation, an additional RF-power pulse was added before each stimulation pulse. The duration of this RF-power pulse phase was 144 and 25 µs, in DS1 and DS2 respectively. The 144 µs phase width of the power-up pulse, as used in the protocol of DS1, was more than sufficient to provide the implant with sufficient power throughout the whole stimulation sequence. This was not the case for the 25 µs phase width as used in DS2 for subject S5. As a result, the first pulses in a single epoch had a lower stimulation level compared to those occurring later in the epoch. Therefore, these pulses were removed prior to the EEG analysis (i.e., a shorter epoch duration was used in the analysis to overcome this problem, see Supplementary Table I). See Gransier et al.^30^ for more specific details about this specific power issue. Due to the differences in clock rate between NIC3 and NIC4 the actual stimulated rates differed slightly from the intended ones. For illustrative purposes and clarification we report the intended/rounded stimulation rates.

### EEG recording

EEG was recorded with an eight-channel Biosemi ActiveTwo HyperRate system. This EEG-recording system had a sample rate of 262.144 kHz per channel^30,39^, which was designed to optimally sample and minimize distortion of the stimulation artifacts. The built-in analog third-order antialiasing filter had its − 3 dB point at 50 kHz. We used this high-bandwidth and high-sampling system as we hypothesized that these system specifications would be sufficient to create accurate artifact templates that can be used on single-trial data, and for recordings where the stimulation is not synchronized with the sampling of the EEG recording device.

We used Ag/AgCl active recording electrodes that were placed on the participant’s head according to the 10–20 system^52^ with the use of a cap. Six pin electrodes were placed on the cap at the locations: P9, P10, Iz, Cz, Fz, and Fpz, and two flat electrodes were placed at the left (MaL) and right mastoid (MaR). The DRL and CMS electrodes were placed near the central parietal-occipital location of POz. Recording electrodes were not attached at sites corresponding to the location of the coil of the CI.

The EEG recordings were done in an electrically-shielded (i.e., Faraday cage) sound booth. Participants were awake, sat in a comfortable chair during the EEG recordings, and watched a captioned silent movie displayed at a screen in front of them. To minimize the effect of muscle and movement the head was supported with a cushion, and participants were asked to move as little as possible during the recording. 

### EEG analysis

**Figure 1:**
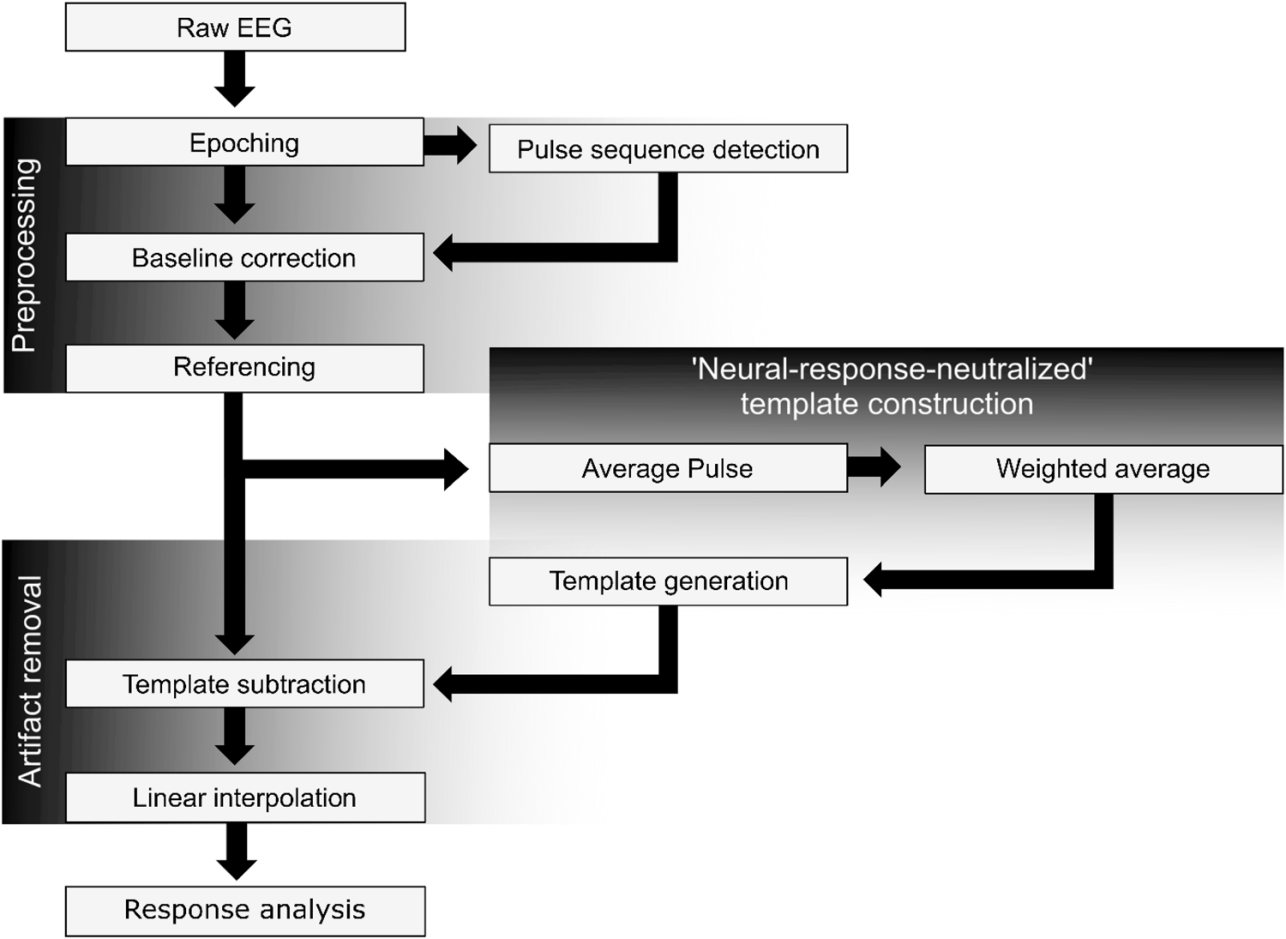
Block diagram of the EEG processing pipeline used to obtain the artifact-free electrically-evoked frequency following responses.

All EEG signal processing was done offline in Matlab R2016B^51^ and statistics and visualizations were made with R^53^. The raw EEG data for each pulse rate, as recorded with the hyper-rate EEG system, were divided into epochs according to the recorded triggers and were stored in a 3D-matrix (${A}_{{Raw}_{(t, E,C)}})$ that was organized based on time ($t$), epoch ($E$), and channel ($C$). The analysis pipeline, as depicted in Fig. 1, consists of the preprocessing stage, a template construction stage, the artifact removal stage, and finally the eFFR analysis, all described below.

#### Preprocessing

##### Pulse sequence detection

First, the start of each pulse within an epoch was determined. This was done to (i) account for small differences in the clock rate between the NIC3 and NIC4 research interface, as this resulted in small deviations between the intended pulse rate and the actually stimulated pulse rate when the NIC4 research interface was used, and (ii) to have the best possible alignment of all the individual pulses when creating the averaged pulse for the construction of the artifact templates (see *neural-response-neutralized artifact template construction*). Given that we are only interested at this stage in identifying the start of each pulse in the EEG recording (i.e., the relative differences in the EEG), we baseline corrected each epoch by subtracting the average amplitude of each epoch from the time series within an epoch according to Eq. (1),

1 $${A}_{{Mean\, corrected}_{(t, E,C)}}= {A}_{{Raw}_{(t, E,C)}-}\frac{1}{N} \sum_{t=1}^{i=N}{A}_{{Raw}_{(t, E,C)}}$$

With the amplitude of the raw EEG signal (${A}_{Raw}$) at each time point $(t)$, for epoch (*E)*, and channel (*C*). Thereafter, the averaged epoch $({\overline{A} }_{{Mean\, corrected}_{\left(t, C\right)}})$ was computed and the start of each pulse within ${\overline{A} }_{{Mean\, corrected}_{(t, C)}}$ was automatically detected based on the rise time by using the built-in *Matlab* function *risetime*. Only amplitudes above a specific cut-off value, which was normally set at 50 µV but could be adjusted if needed, were taken into account. This was done to ensure that the RF-pulse and other unwanted transients did not affect the detection of the detection of the actual stimulated pulses. We used the recording electrode positioned at the ipsilateral mastoid (i.e., the channel with largest artifact) to determine the timepoint of the start of each pulse within the stimulated pulse train per epoch; in the following we refer to this sequence of start points as the pulse sequence.

##### Baseline correction and referencing

**Figure 2:**
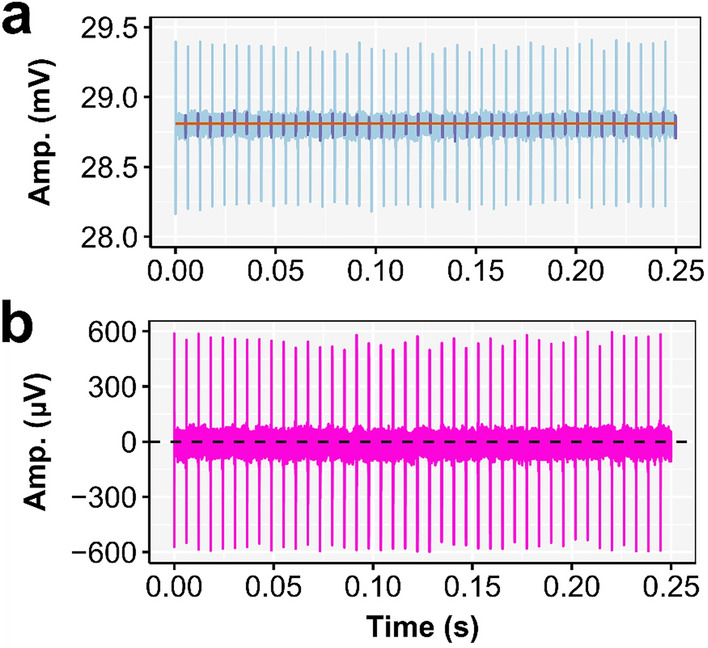
Illustration of the baseline correction. (**a**) The raw EEG data ${(A}_{Raw}$) of CI user S4 when stimulated with a pulse train of 163 pps. The EEG is recorded with the EEG electrode located at the right mastoid (MaR; light blue) and only a part of the second epoch is shown. All EEG datapoints between 80 and 85% of the inter-pulse interval (purple) were used to fit the baseline signal (orange). (**b**) The baseline corrected EEG $({A}_{cor})$ as recorded with MaR.

Next, ${A}_{{Raw}_{(t, E,C)}}$ was baseline corrected. The baseline signal was constructed per channel and epoch, based on the linear regression coefficients that were fitted to the data, at the timepoints between 80 and 85% of each inter-pulse interval of all consecutive pulses (Fig. 2a). This baseline signal was then subtracted from the raw EEG signal according to Eq. (2).

2 $${A}_{{cor}_{(t, E,C)}}= {A}_{{Raw}_{\left(t, E,C\right)}}-({\alpha }_{\left(E,C\right)}+ {\beta }_{\left(E,C\right)}\cdot t)$$

With $\alpha$ and $\beta$ as the individual fitting parameters per epoch $(E)$ and channel $(c)$. This way of baseline correction was chosen as (i) it enables a correct representation of the stimulation artifact when using an averaged single pulse to create the artifact template, and (ii) it does not introduce a DC component—as is the case when using the approach as in Eq. (1)—which can differ across epochs and channels (e.g., due to the recording site dependent differences in asymmetry of the recorded stimulation artifact). We empirically selected the 80–85% of the inter-pulse timepoints used in the linear regression because this was at the end of the decaying artifact and did not include the RF pulses that were added before each stimulation pulse to provide the implant with enough power for stimulation. As a result, the DC differences between recording electrodes were minimized prior to offline referencing. The baseline corrected EEG (${A}_{{cor}_{(t, E,C)}}$) (Fig. 2b) was used in the further analysis and for the construction of the artifact template.

**Figure 3:**
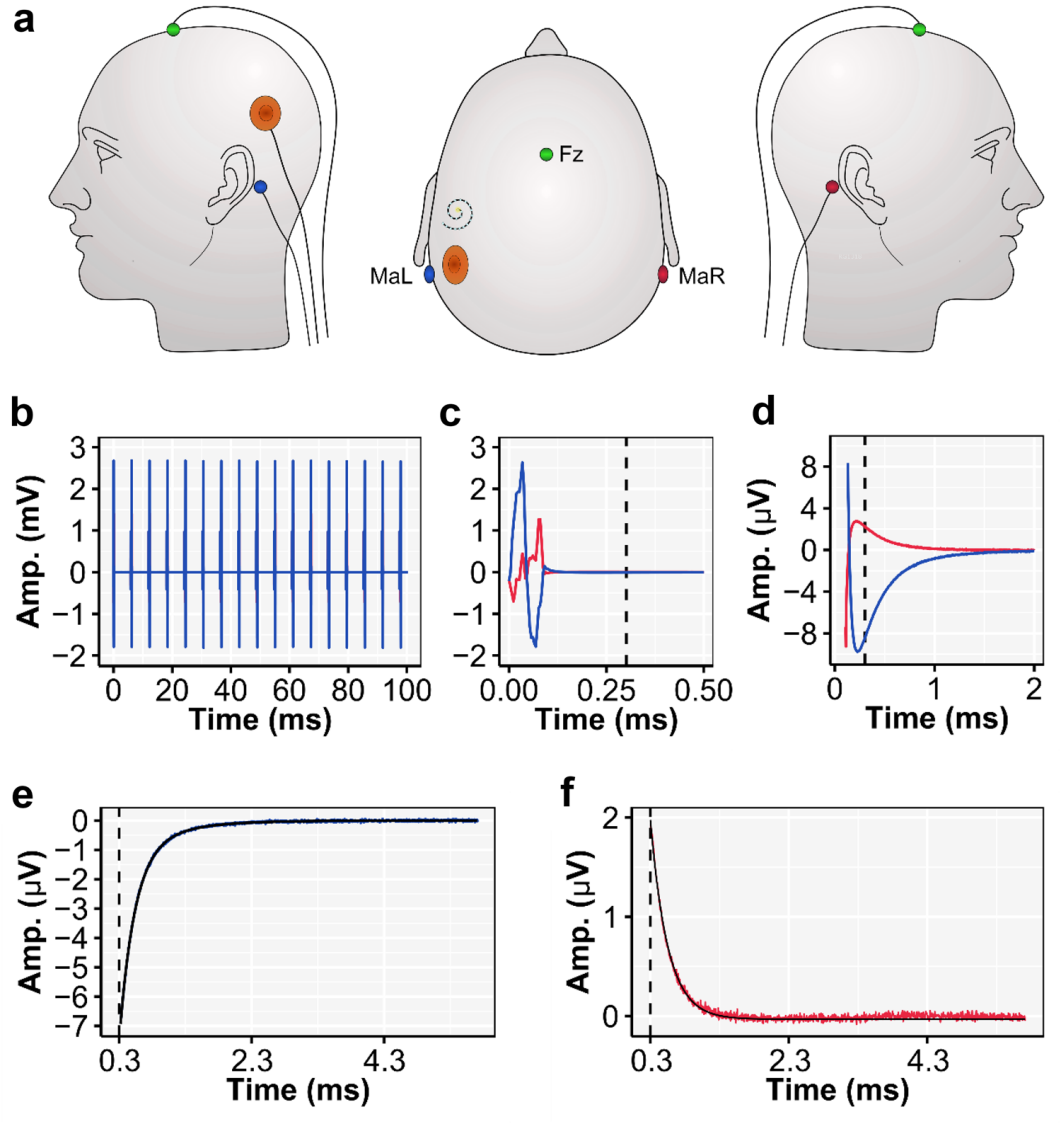
(**a**) Illustration of the EEG electrode placement on the head of cochlear-implant (CI) user S4. EEG electrodes were placed at the left (MaL) and right mastoid (MaR) and were referenced to the EEG electrode placed at Fz. The CI was implanted in the left cochlea and the RF coil is shown in orange. (**b**) The first 100 ms of an average epoch (${A}_{{cor}_{ref}}$) of both the ipsi and contralateral mastoid, i.e., MaL (blue) and MaR (red) respectively. The stimulation artifact of the 163 pps pulse train is clearly present in the EEG and note that no neural response was evoked in this specific case. (**c**) The amplitude zoom of the first 0.5 ms the grand-averaged single pulse waveform (${\overline{A} }_{{cor}_{pulse}}$) showing predominantly the biphasic stimulation artifact and superimposed RF artifact, and (**d**) the amplitude zoom 0.05–2 ms of the same single pulse stimulation artifact showing the artifact tail that starts after 0.3 ms (indicated with the dashed line). The amplitude zoom of the exponential tail (${\overline{A} }_{{cor}_{pulse}}$) and the fitted artifact model (${{\text{A}}}_{{\text{tail}}}$; solid black line) for both the ipsi (**e**) and contralateral channel (**f**). Note that the pulse train in this example, presented at MCL, did not evoke an eFFR and therefore consists only of the stimulation artifact and the residual neural noise. The grand-averaged single-pulse artifact in (**c**–**f**) is based on 185,704 pulses. The dashed line in (**c**–**f**) shows the start of the artifact tail which was used to fit the artifact tail model.

**Figure 4:**
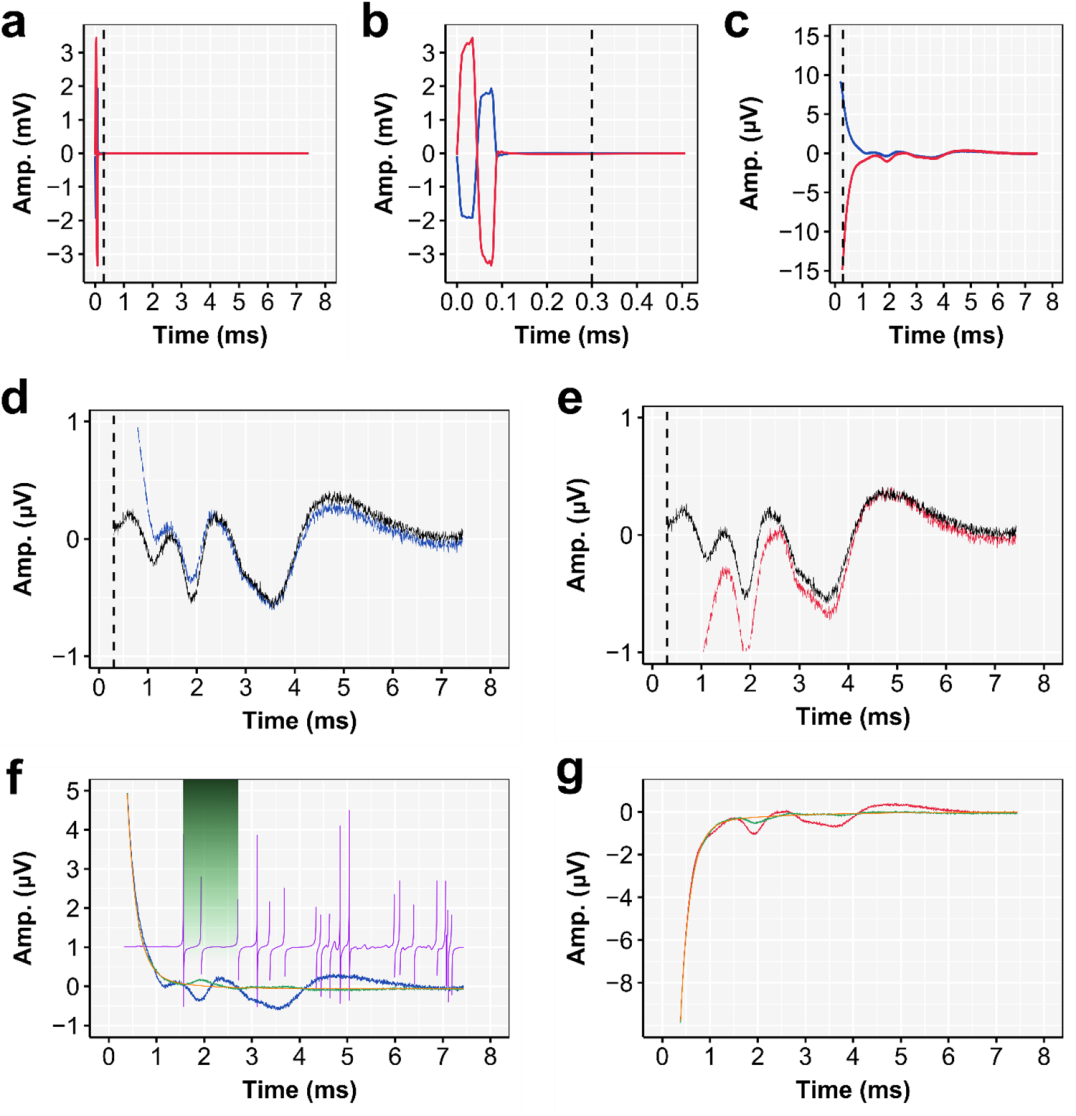
Example of neural- with overlapping artifact response characteristics. Data shown is from subject S1 and for the 128 pps condition. (**a**) The grand-averaged single pulse waveform (${\overline{A} }_{{cor}_{pulse}}$), containing both the stimulation artifact and the eFFR, recorded with EEG electrodes were placed at the left (MaL; in blue) and right mastoid (MaR; in red) referenced to the EEG electrode placed at Fz. (**b**) The first 0.5 ms of the grand-averaged single pulse waveform, showing biphasic stimulation artifact and (**c**) a zoom showing the section from the start of the tail (dashed line) to the end of the inter-pulse interval. (**d**,**e**) The response tail (${\overline{A} }_{{cor}_{pulse}}$) and overlapping eFFR as recorded at MaL (d, blue line) and MaR (e, red line) and the weighted average (${\overline{A} }_{{Neural}_{approx}}$; shown in black in (**d**) and (**e**)). (**f**) The response tail as recorded at MaL (${\overline{A} }_{{cor}_{pulse}}$; blue), the approximation of the artifact tail (${\overline{A} }_{{Tail}_{approx}}$; green) and the artifact model fit (${{\text{A}}}_{{\text{tail}}}$; orange). The derivative factor (Eq. 7) is shown for illustrative purposes in purple and the section of the artifact approximation that contains the dissimilarity is shown by the green shaded area. (**g**) The artifact tail as recorded at MaR (red), the approximation of the artifact tail (green) and the artifact model fit (orange).

After baseline correction, all channels were referenced to Fz (${A}_{{{cor}_{ref}}_{(t, E,C)}}$) and only the active electrodes positioned at the left (MaL) and right mastoid (MaR) were used in the analysis (see Fig. 3a). These vertical montages (MaL-Fz and MaR-Fz) (Fig. 3a) were chosen as they optimally capture the dipole orientation from the brainstem^54^. We especially chose Fz as a reference electrode, as this generally records a lower-magnitude stimulation artifact compared to more central-occipital located electrodes (e.g., Cz), while at the same time contains lower noise levels—due to eye movements and muscle artifacts—compared to more central-frontal located artifacts (e.g., Fpz). Furthermore, these two vertical montages contain stimulation artifacts that are in opposite phase from each other (Fig. 3b–d), whereas they have similar in-phase neural responses across recording sites (Fig. 4). These differences in artifact and similarities in response characteristics, as will be clear in the following, are essential for the construction of the neural-response-neutralized artifact templates. Given that different ears were tested across participants, we will refer in the following only to the location of the active recording electrode relative to the location of the CI (i.e., the ipsilateral or contralateral channel).

#### Template construction

After preprocessing, the neural-response-neutralized artifact template of the artifact tail was constructed, the RF pulse and biphasic pulse are removed in a later stage by means of linear interpolation as these cannot be modeled properly. We first segmented all individual pulse intervals from ${A}_{{{cor}_{ref}}_{({T}_{i}, E,C)}}$ based on the start positions determined previously from the pulse sequence. All individual pulses were then averaged, resulting in ${\overline{A} }_{{{cor}_{pulse}}_{(t, C)}}$. The minimum amount of pulses that were included in the average depended on the pulse rate and was 85,737 and 240,904 pulses, for respectively 128 and 303 pps (see Supplementary Table I). Figure 3c,d shows the different sections of a single-pulse stimulation artifact recorded with the ipsi and contralateral channel in a representative subject with no measurable eFFRs (i.e., artifact-only data). The stimulation artifact derived from ${\overline{A} }_{{{cor}_{pulse}}_{(t, C)}}$ is characterized (i) by an artifact that is in opposite phase when recorded with the ipsi- compared to the contralateral channel, (ii) the artifact recorded with the contralateral channel is of a much lower magnitude compared to the artifact recorded with the ipsilateral channel, and (iii) the stimulation artifacts recorded with both the ipsi- and contralateral channel have tails that decay exponentially beginning after approximately ~ 0.3 ms (i.e., 80 samples) of the pulse start; the 0.3 ms time point in each plot is denoted by the vertical dashed line. This tail (${\overline{A} }_{{{cor}_{Tail}}_{(t, C)}}$) can be modeled with two decaying exponentials (Fig. 3e,f, Eq. 3).

3 $${{\text{A}}}_{{{\text{tail}}}_{{\text{t}}}}= \gamma \cdot {e}^{\left(\delta \cdot t\right)}+ \varepsilon \cdot {e}^{\left(\zeta \cdot t\right)}$$

With ${A}_{{tail}_{t}}$ as the artifact-tail model at timepoint *t,* starting from 0.3 ms after the start of the biphasic pulse, and $\gamma , \delta ,\upvarepsilon$, and $\upzeta$ as the individual fitting parameters. This was calculated with the built-in Matlab *Fit* function.

In cases when a neural response time-locked to the pulse train is present (e.g., the eFFR), the recorded tail at time point *t* will contain both artifact and neural response (Fig. 4). As a result, the artifact tail cannot be properly modeled because the model will be the best fit to the combination of both the artifact tail and the time-locked neural response. To overcome this issue, we remove the neural response from the artifact tail, so that only the ‘true’ artifact tail remains. In this neural-response neutralization step, an approximation of the neural response is computed by a weighted average of the ipsi- and contralateral channel (Eq. 4). Due to the fact that signals recorded with the ipsi- and contralateral channels have in-phase response characteristics but out-of-phase artifact characteristics (Figs. 3, 4), this weighted averaging of the two channels will cancel-out the artifact. This approach is similar to the averaging approach often used to average out an artifact component by averaging two stimulation polarities^42,43^, but with the advantages that no additional data need to be collected to construct the artifact templates, and that the neural response -after artifact subtraction- is not affected by any differences in neural responsiveness as can be the case when different stimulation polarities are used^41,46–48^.

4 $${\overline{A} }_{{{Neural}_{approx}}_{(t)}}= \frac{1}{{N}_{ipsi}{+ N}_{contra}} \cdot \left(\sum_{p = 1}^{{ N}_{ipsi}}{A}_{{{cor}_{Tail}}_{(t, p,{C}_{ipsi})}} +\sum_{p = 1}^{{ N}_{contra}}{A}_{{{cor}_{Tail}}_{(t, p,{C}_{contra})}}\right)$$

With *p* as the individual pulse, and ${N}_{ipsi}$ and ${N}_{contra}$ as the number of pulses included in the weighted average for respectively the ipsi ${(C}_{ipsi})$ and contralateral channel ${(C}_{contra}$). The number of pulses used, per channel, in the weighted average is determined by the factor difference (Eq. 5).

5 $${Factor\, Difference}_{(t)}= \frac{\left|{A}_{{MaL}_{(t)}}\right|}{\left|{A}_{{MaR}_{(t)}}\right|}$$

With $A$ as the amplitude at time $t$. The factor difference between the in-phase or opposite-phase artifacts, in cases when no response and neural noise present, is approximately constant across the whole artifact tail (Supplementary Fig. 1). However, this is not the case when a neural response or a large amount of neural noise is present. Therefore, we used the mean of the first two samples (i.e., ~ 0.3 ms) of the artifact tail (i.e., the peak of the tail and therefore the most artifact dominated part of the tail) to determine the channel-specific number of single-stimulation pulses, rounded to the nearest integer, to include in the weighted average (Eq. 4). Figure 4 and Supplementary Fig. 1 show examples of the weighted-average approach in cases in which a neural response was present or absent, respectively. All weighted averages are shown in Supplementary Fig. 2. Note that the weighted-average is biased towards the eFFR recorded with the channel contralateral to the CI, as this has the smallest artifact and therefore the largest number of pulses included in the weighted average.

We then applied the neural-response neutralization to obtain an unbiased and channel-specific artifact tail ${\overline{A} }_{{Tail}_{{approx}_{(t,C)}}}$ approximation by subtracting ${\overline{A} }_{{{Neural}_{approx}}_{(t)}}$ from the channel specific recorded tail ${\overline{A} }_{{{cor}_{Tail}}_{(t, C)}}$. Neural-response neutralization was only done when a neural response was deemed to be present. The presence of a neural response was based on a composite score: the neural index (NI), which takes into account the characteristics of the weighted average time series. The NI is based on the variance, range, and skewness of the time series’ distribution (Eq. 6). The reasoning behind the use of the composite score is that when no neural response is present the distribution of the amplitude values within the weighted-average waveform is gaussian, with a relatively small variance. In cases in which a neural response is present, the distribution of the amplitude values within the weighted-averaged time series becomes more skewed (as denoted by S; the skewness) and the variance (as denoted by ${\sigma }^{2}$) increases (Supplementary Fig. 3).

6 $$NI= 1000\cdot \left|{\text{max}}\left({A}_{{t}_{i}=1,{t}_{i}}\right)- {\text{min}}({A}_{{t}_{i}=1,{t}_{i}})\right| \cdot {\sigma }^{2} \cdot \left|S\right|$$

Neural-response neutralization was only applied when the NI was > 0.4. Any score below this cut-off was indicative of the absence of a neural response or a neural response so small that it would not affect the fit of the artifact model. This cut-off value was empirically determined and based on the datasets used in the present study (Supplementary Fig. 4).

One of the disadvantages of subtracting the weighted average, as described above, is that it is biased towards the contralateral channel, i.e., the channel with the lowest artifact magnitude. Whenever there is a dissimilarity between the neural responses recorded with the ipsi- and contralateral electrodes, this dissimilarity is reflected in the weighted average. When such a dissimilarity is present, ${\overline{A} }_{{Tail}_{{approx}_{(t,C)}}}$ will contain a neural response component that can affect the artifact model fit. To detect the presence of a dissimilarity, which potentially could affect the artifact modelling, we computed the derivative factor per channel (Eq. 7).

7 $${Derivative\, factor}_{(t)}=\frac{{A}_{{Tail}_{{approx}_{(t)}}}-{A}_{{Tail}_{{approx}_{(t+1)}}}}{{A}_{{Tail}_{{approx}_{(t+1)}}}-{A}_{{Tail}_{{approx}_{(t+2)}}}}$$

In cases in which no neural response is present (i.e., an exponential decaying artifact only) then the derivative factor will be approximately constant (i.e., approximately unity). However, if a neural response is present, then there will be a deviation from the double exponential decay and this is characterized by a positive and negative deflection in the derivative factor at timepoint *t* where the deviation occurs (see purple line in Fig. 4f). When deviations are detected we excluded the data between the first three deflections from the tail data of $${A}_{{Tail}_{approx}}$$ to fit the artifact model according to Eq. (3) (Fig. 4f,g). The data between the first three deflections correspond, as is clear from the results, with the peak III of the eFFR waveform. This approach enables a channel-specific unbiased fit of the artifact model ($${A}_{tail})$$. Finally, the channel-specific artifact-tail model is created at the epoch level by concatenating the artifact tail model over the entire pulse sequence.

#### Template subtraction and linear interpolation

The constructed artifact tail model sequence, at epoch level, was then subtracted from each individual epoch in ${A}_{{cor}_{(t, E, C)}}$ and the RF burst prior to the biphasic pulse and the actual biphasic pulse were removed by means of linear interpolation^40^. An interpolation interval of only 100 samples after the start of each pulse (0.0–0.38 ms) was used, resulting in the artifact free EEG (${A}_{{Artifact\, free}_{(t, E, C)}})$.

#### Response analysis

After artifact removal, 5% of the epochs with the highest amplitudes were removed and a Fast Fourier Transform was done at epoch level. The mean response amplitude and phase were computed by vector averaging the complex response spectrum across epochs. The neural background noise was calculated as the standard deviation over epochs divided by the square root of the number of epochs^55^. A Hotelling T^2^ test^40,56^ was used to determine if a significant eFFR was present, per channel, at the fundamental frequency (i.e., the first harmonic) and/or at the second harmonic. A significance level of 1% was used to account for multiple comparisons and the relatively low noise levels associated with the frequency ranges used here (128–600 Hz).

The latency of the eFFRs was based on the group delay across response frequencies at which a significant neural response was detected. To obtain the latency, the phase slope—which was determined by a linear regression fitted to the unwrapped phase of the eFFRs across the different pulse rates—was divided by 360 degrees^57^. In the case that the artifact removal, as described above, is not successful the latency would be 0 ms^30,32,40^. We therefore also use the latency to evaluate the effectiveness of the neural-response-neutralized based artifact removal.

## Results

**Figure 5:**
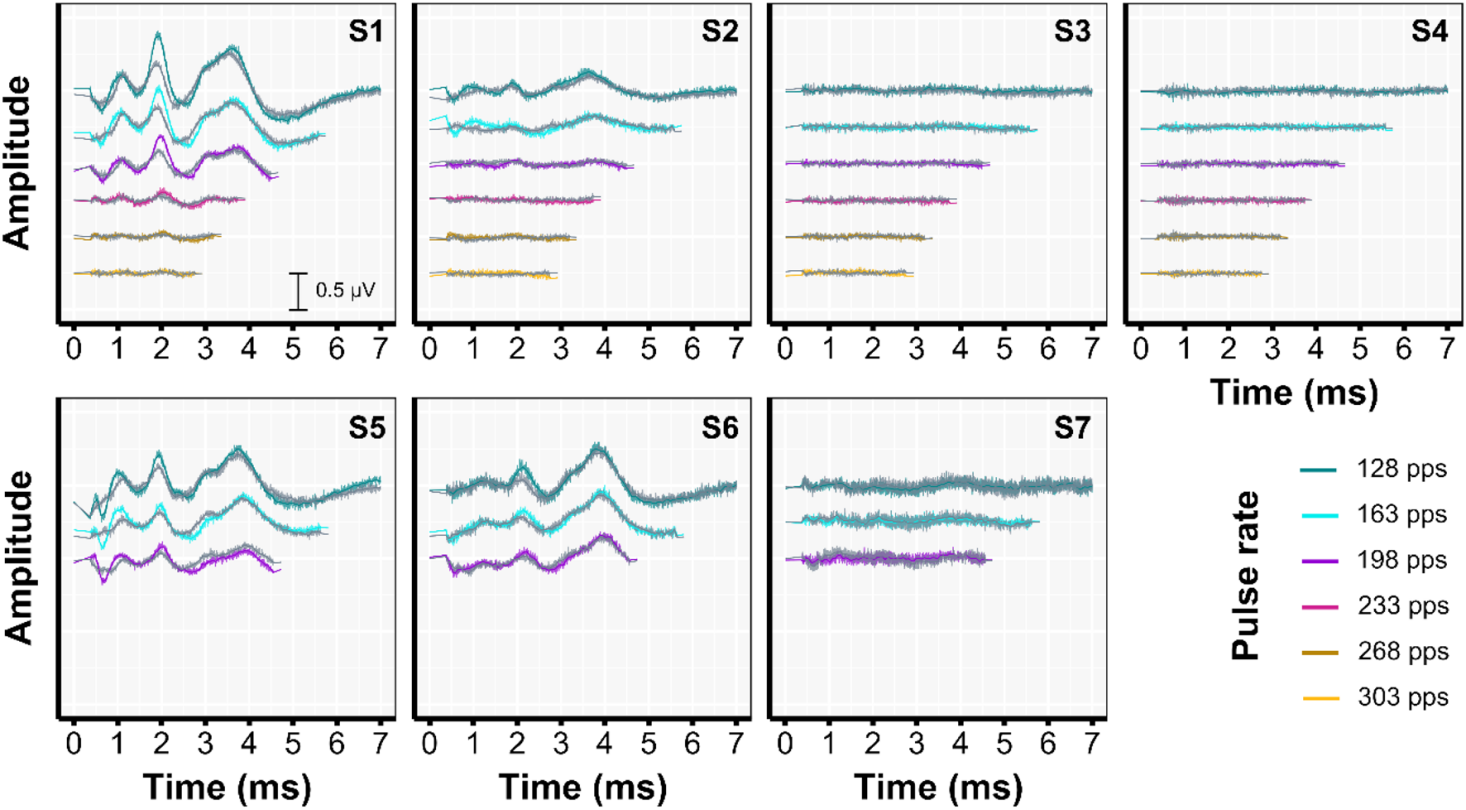
Individual grand-averaged single-pulse neural response waveforms after artifact removal for the ipsi and contralateral electrodes. The waveform, as recorded with the ipsilateral electrode is shown in the color of the corresponding pulse rate and the contralateral electrode in gray. These single-pulse averages were computed by averaging all individual inter-pulse intervals of the artifact free EEG (${A}_{Artifact\, free})$. Due to the different pulse rates, i.e., different inter-pulse intervals, the duration of the neural responses, as shown, differs between pulse rates.

Figure 5 shows the averaged single-pulse waveforms after artifact removal. Four of the seven participants had time-locked neural responses that were observable for pulse rates up to 196 or 198 pps, and of the four participants tested at higher rates, only S1 showed visible peaks up to 233 pps. The time-locked neural responses were characterized with peaks at 1, 2, and ~ 3.5 ms and correspond to the eABR peak II, peak III and peak V respectively^41,49^. Although peak I could not be observed due to the 0.38 ms blanking length, its trough is clearly observable. With increasing pulse rate the peaks became overall smaller in magnitude and the latency increased slightly. Peak III remained the most visible with increasing pulse rate. Waveforms recorded with the ipsi and contralateral electrodes were highly similar with the exception of peak III which was higher in magnitude in the ipsilateral compared to the contralateral recorded waveform.

**Figure 6:**
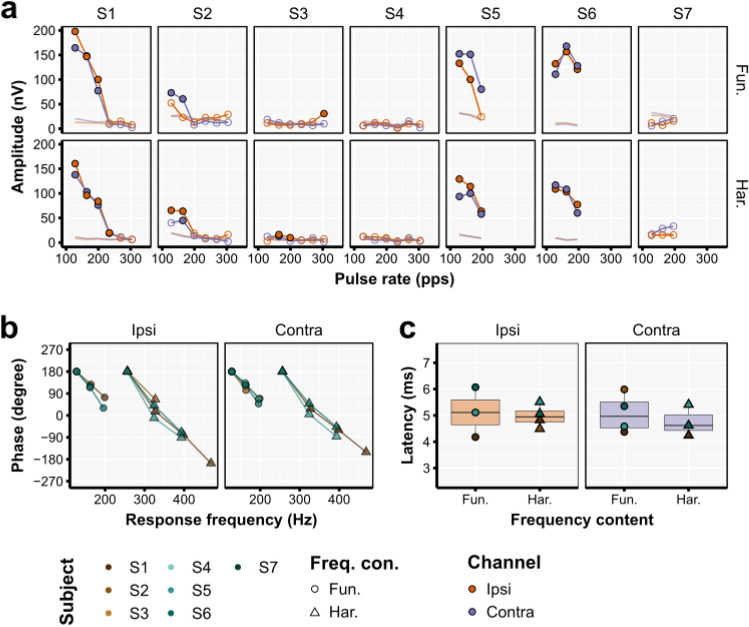
(**a**) The eFFR amplitude as a function of pulse rate for the fundamental frequency (first harmonic; Fun.) and the harmonic (i.e., second harmonic; Har.) as derived from the artifact-free EEG (${A}_{Artifact\, free}$). (**b**) The response phase as a function of the response frequency. Only the data of the participants that had more than two significant responses are shown. The data of participant S2 for the second harmonic was omitted due to the low response magnitude values. (**c**) The latencies derived from the phase slope.

Spectral analysis of the eFFRs at epoch level, show that the CI users could be clearly divided into two groups: those who did not have an eFFR present at either the fundamental or the harmonic frequency (participant S3, S4, and S7) and those in which eFFRs could be measured (participant S1, S2, S5, and S6). Overall the response strength within a participant was similar between the fundamental and harmonic response frequency and between the ipsi and contralateral channel (Fig. 6a). The maximum eFFR amplitude was 197.8 nV, which was evoked with the 128 pps pulse train. There is a clear reduction in eFFR magnitude with increasing pulse rate, and the maximum pulse rate at which an eFFR component could still be detected was as high as 233 pps (i.e., Participant S1). The neural background noise levels were extremely low and had the standard 1/f shape with increasing frequency (Supplementary Fig. 5). At average noise level across subjects and electrode sites was 19.5 nV (SD = 9.2 nV) at 128 Hz and 5.5 nV (SD = 1.0) at 608 Hz.

The phase delays showed that there was a consistent phase lag across response frequencies (Fig. 6b). The corresponding latencies, for both the ipsi- and contralateral channel were between 4.18 and 6.07 ms, with an average latency of 4.99 ms (SD = 0.62 ms) (Fig. 6c) and were consistent with that of a brainstem generator^58^. The obtained latencies, in combination with the non-significant responses and low noise levels indicate that the template subtraction with neural-response-neutralized template construction was effective in removing the stimulation artifacts of the CI stimulation from both the ipsi- and contralateral channel.

## Discussion

The main difficulty of measuring electrophysiological responses during continuous electrical stimulation is the separation of the neural response from the stimulation artifact^59^. Many artifact-removal methods aim to reduce the artifact to such an extent that the neural response of interest becomes visible. Although this approach works well when the aim is to attenuate the artifact to such a degree that one can visually detect a neural response, as when there is a large neural response magnitude compared to the artifact (e.g., the eCAP, the peak V of the eABR, or cortically evoked onset response), these methods fail when the artifact-to-response ratio is large and/or when the artifact and neural response overlap both in the time and frequency domain. In case of the eFFR, both difficulties apply. Here we introduced a template subtraction artifact-removal method that is able to separate the stimulation artifact tail from the neural response, by first removing an estimate of the neural response from the recordings, which in turn enables the generation of an artifact model that is not affected by the neural response. As a result, this method is able to fully separate the artifact from the neural response, even though both fully overlap in the time and frequency domain.

The approach we took to construct the artifact templates is counter intuitive. Most conventional artifact removal methods, including those that use template subtraction, aim to directly remove the stimulation artifact from the recording^41,45,49^. However, this poses difficulties when the neural response coincides with artifact, especially when the latter is orders of magnitude larger than the former. As a result, an artifact template model will be based on a combination of both the artifact and the neural response. By using the neural-response-neutralization step prior to constructing the artifact template, we were able to isolate the stimulation artifact from the neural response and thereby create an unbiased channel-specific artifact model.

The neural-response-neutralization approach, aided by the large amount of averages and high-sampling rate, enabled insight in the true shape of the artifact tail. The stimulation-artifact tail could be modelled, depending on the polarity, with two increasing or decaying exponentials. The validity of this artifact model is clear from the complete removal of the stimulation artifact in the rate-conditions that did not result in a neural response and the fact that in some participants no eFFRs could be detected (see Fig. 5 and Supplementary Fig. 1).

Although the primary aim of our research was to obtain artifact-free eFFRs, it is clear from the single-pulse waveforms (Fig. 5) that our artifact removal method can also be used to obtain artifact-free eABRs over a range of rates much faster than commonly employed using conventional techniques. Only the first 0.38 ms—in which the peak I of the eABR occurs—is omitted due to the removal of the biphasic pulse artifact by linear interpolation. As a result this method is able to, in combination with eCAP measures, provide a full insight in the neural responsiveness from the most distal parts of the auditory nerve up to the brainstem and the higher structures of the electrically-stimulated auditory pathway. We observed that with increasing pulse rate the later peaks in the single-pulse waveform disappear and the overall amplitude of the remaining peaks decreases. Whether this is affected by the refractory period of the auditory nerve or due to neural adaptation, which can both interact with the pulse rate^44^, cannot be assessed with the current data.

The eFFR was characterized by frequency components at both the fundamental frequency and the first harmonic. Whenever eFFRs could be elicited, the amplitudes of both decreased with increasing pulse rate. Although not all rates were presented to all participants, the maximum rate at which an eFFR could be detected was 233 pps. The exception to this is the eFFR detected at 303 pps in participant S3, however, this is probably a false positive, as no consistent eFFRs could be evoked in that participant for lower rates. That the template subtraction method is effective in removing the stimulation artifacts is also clear from the phase delays and the derived apparent latencies, which were on average 5 ms and are consistent with a predominant brainstem generator and is also similar to the latency observed in the acoustically evoked FFR in normal-hearing listeners^58^. Although the sample size of the present study is low and not all rates were tested in all subjects, it is of interest that only in half of the participants eFFRs were detected. In case eFFRs were present, especially for rates < 200 pps, the amplitudes were similar across the participants. In contrast, upper limit of phase-locking to electrical pulse trains, as reported here, is much lower than that of normal-hearing listeners when listening to acoustical pulse trains^60^. How the phase-locking ability of the brainstem of CI-users relates to functional performance is currently unknown, but the present study clearly demonstrates that the here introduced artifact removal method enables to investigate this in future studies.

One of the advantages of the neural-response-neutralized template construction is that it does not require any additional measurements, such as is often the case with other template-based artifact removal methods^33,42,61^. The here introduced method is not only more time-efficient, but it also does not need any additional measures, e.g., stimulating in alternating polarity, and therefore also enables the investigation of stimulation polarity effects on the brainstem response^41,46–48^. Furthermore, the template subtraction methods introduced here can potentially be used to remove the stimulation artifacts in other stimulation paradigms, as long as the neural response has in-phase and the artifact out-of-phase artifact characteristics between two electrode configurations that are used in the neural-response-neutralization step.

To summarize, the template subtraction method, based on neural-response-neutralized template construction, enables the full removal of the stimulation artifact, and in particular when the neural response and stimulation artifact overlap in both the time and frequency domain. Although a high-sampling EEG system is required to optimally sample the stimulation artifact, the template construction method is a relatively simple algorithm which makes optimal use of the response and artifact characteristics and has the potential to be implemented in clinical EEG recording devices not only to measure artifact-free eFFRs but also other electrophysiological responses such as the eABR.

### Supplementary Information

Supplementary Information.

## Data Availability

Data is available from the corresponding author upon reasonable request.
