# Supplementary material for: Artifact removal by template subtraction enables recordings of the frequency following response in cochlear-implant users
Source: Sci Rep. 2024 Mar 14;14:6158. doi: 10.1038/s41598-024-56047-9 (PMC10940306; doi:10.1038/s41598-024-56047-9)
Supplement: Supplementary file 1 — Supplementary Information. [file 41598_2024_56047_MOESM1_ESM.docx]

**Artifact removal by template subtraction enables recordings of the frequency following response in cochlear-implant users**

**Supplementary material**

Robin Gransier^1^, Robert P. Carlyon^2^, Matthew L. Richardson^3,4^, John C. Middlebrooks^3,4.5^, and Jan Wouters^1^

^1^ ExpORL, Department of Neurosciences, Leuven Brain Institute, KU Leuven, Leuven, Belgium.

^2^ Cambridge Hearing Group, MRC Cognition and Brain Sciences Unit, University of Cambridge, Cambridge, UK.

^3^ Department of Otolaryngology, University of California at Irvine, Irvine, CA, USA.

^4^Center for Hearing Research, , University of California at Irvine, Irvine, CA, USA.

^5^ Departments of Neurobiology & Behavior, Biomedical Engineering, Cognitive Sciences, University of California at Irvine, Irvine, CA, USA.

Corresponding author: jan.wouters@kuleuven.be

**
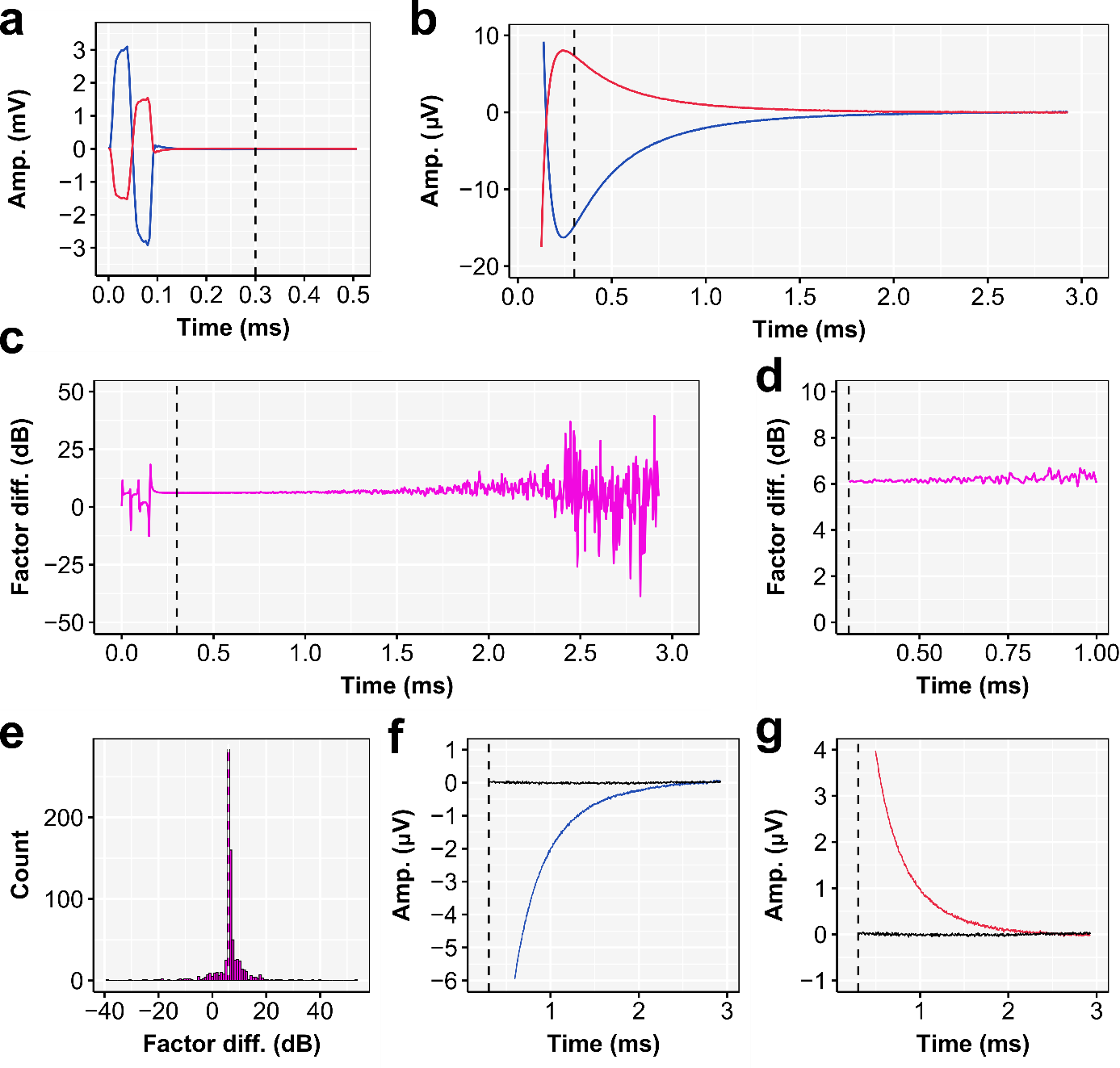
**

**Supplementary Figure 1 |** Illustration of weighting average approach in case no neural response is present. Data shown is from participant S2 and for the 303 pps condition. **(a)** The first 500 µs of the grand-averaged inter-pulse interval ($\bar{A}_{{cor}_{pulse}}$) of both the ipsi and contralateral mastoid (i.e., MaL (Blue) and MaR (Red)). **(b)** the zoom of the artifact tail of the same single pulse stimulation artifact. **(c)** The factor difference as a function of time. **(d)** A zoom of the factor difference that shows that the factor difference is relatively stable over time when no neural response is present. **(e)** The histogram of the factor difference within an inter-pulse interval. The dashed white line shows the average factor difference of the first two samples of the tail, indicating that this is a good approximation to determine the amount pulses that need to be used in the weighted average. **(f-g)** The recorded tail ($\bar{A}_{{cor}_{pulse}}$) in blue or red and the weighted average ($\bar{A}_{{Neural}_{approx}}$) in black.


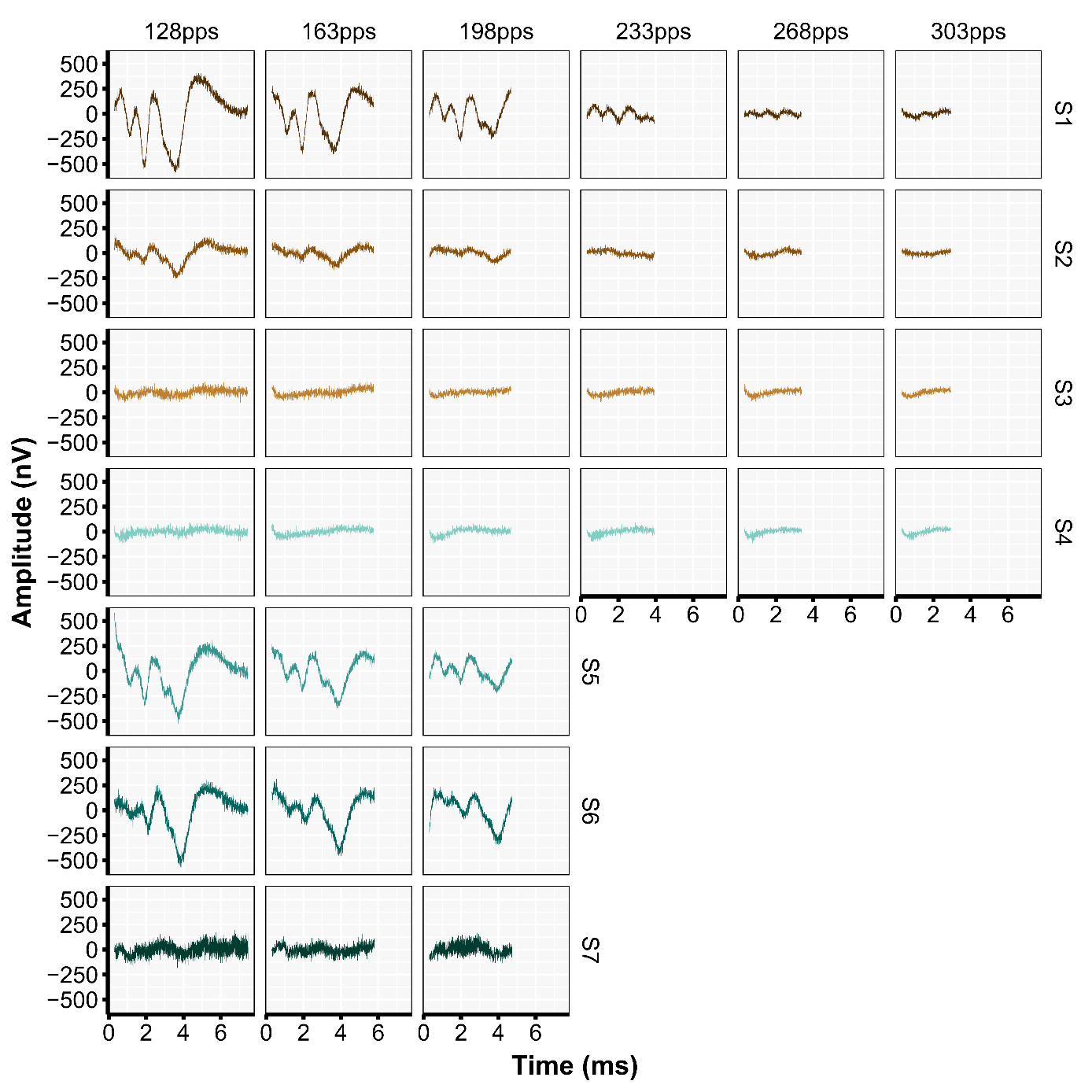


**Supplementary Figure 2 |** The subject-specific (rows) and condition-specific (columns) weighted average $(\bar{A}_{{Neural}_{approx}}$). Note that only one inter-pulse interval is shown.


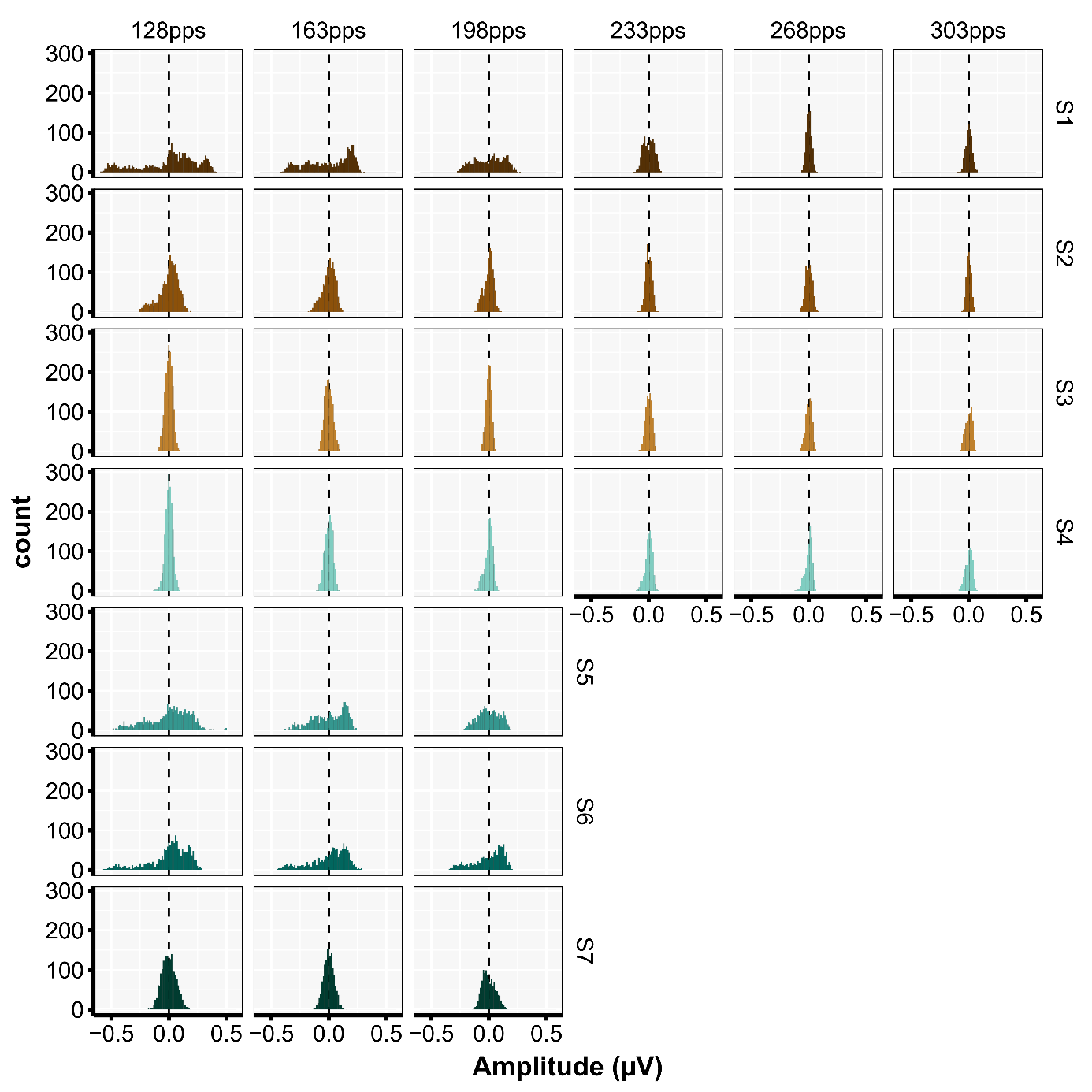


**Supplementary Figure 3 |** The subject-specific (rows) and condition-specific (columns) distribution of the amplitude values in the weighted average $(\bar{A}_{{Neural}_{approx}}$). Note that the distributions are based on one inter-pulse interval.


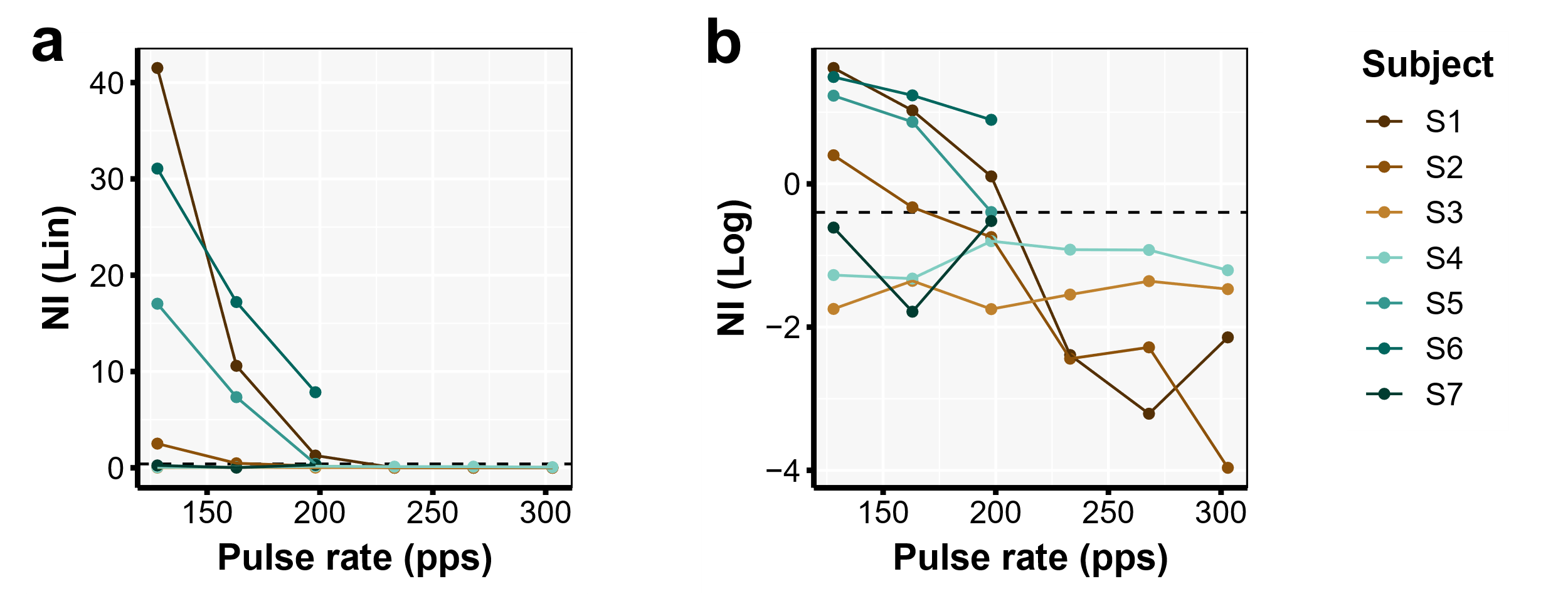


**Supplementary Figure 4 |** The subject-specific neural index as a function of pulse rate on a (**a**) linear scale and (**b**) for illustrative purposes on a log_10_ scale (i.e., the log10(NI linear). The dashed line indicates the threshold that is used to determine if a neural response is present in the weighted average $(\bar{A}_{{Neural}_{approx}}$).


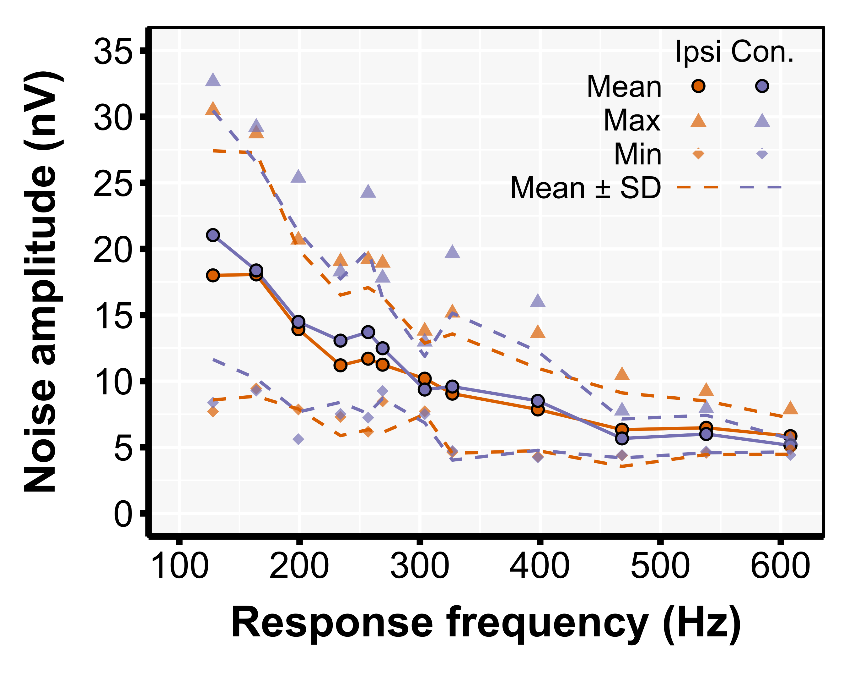


**Supplementary Figure 5 |** The characteristics of the neural noise levels across all participants as a function of the response frequency. Note that the response frequencies which differed minimally between dataset DS1 and DS2 are grouped to compute representative descriptive statistics. Note that the maximum pulse rate differed between DS1 and DS2 and therefore the sample size is lower for response frequencies > 400 Hz.

**Supplementary Table I** | descriptive information about the epochs and number of pulses included in the analyses. The total amount of pulses in the recording is the number on which the average pulse is based, as used in the construction of the artifact template.

| Subject | Pulse rate (pps) | Recorded epochs  (n) | Epoch duration (s) | Total mount of pulses in the recording (n) |
| --- | --- | --- | --- | --- |
| S1 | 128 | 388 | 2.042 | 101433 |
| S1 | 164 | 388 | 2.042 | 129961 |
| S1 | 198 | 388 | 2.042 | 157697 |
| S1 | 234 | 388 | 2.042 | 185433 |
| S1 | 269 | 388 | 2.042 | 213168 |
| S1 | 303 | 388 | 2.042 | 240904 |
| S2 | 128 | 485 | 2.042 | 126792 |
| S2 | 164 | 388 | 2.042 | 129961 |
| S2 | 198 | 449 | 2.042 | 182489 |
| S2 | 234 | 485 | 2.042 | 231791 |
| S2 | 269 | 388 | 2.042 | 213168 |
| S2 | 303 | 449 | 2.042 | 278778 |
| S3 | 128 | 388 | 2.042 | 101433 |
| S3 | 164 | 388 | 2.042 | 129961 |
| S3 | 198 | 485 | 2.042 | 197121 |
| S3 | 234 | 388 | 2.042 | 185433 |
| S3 | 269 | 388 | 2.042 | 213168 |
| S3 | 303 | 485 | 2.042 | 301130 |
| S4 | 128 | 430 | 2.042 | 112413 |
| S4 | 164 | 528 | 2.042 | 176855 |
| S4 | 198 | 485 | 2.042 | 197121 |
| S4 | 234 | 430 | 2.042 | 205505 |
| S4 | 269 | 528 | 2.042 | 290085 |
| S4 | 303 | 485 | 2.042 | 301130 |
| S5 | 128 | 386 | 1.735 | 85737 |
| S5 | 162 | 387 | 1.937 | 121433 |
| S5 | 196 | 387 | 1.972 | 149544 |
| S6 | 128 | 388 | 2.048 | 101711 |
| S6 | 162 | 388 | 2.048 | 128728 |
| S6 | 196 | 385 | 2.048 | 154542 |
| S7 | 128 | 384 | 2.048 | 100664 |
| S7 | 162 | 385 | 2.048 | 127735 |
| S7 | 196 | 384 | 2.048 | 154142 |
